# Serotypic distribution and antimicrobial resistance of *Streptococcus pneumoniae* in Chinese children under 5 years after the introduction of the 13-valent conjugate pneumococcal vaccine: protocol for a scoping review

**DOI:** 10.12688/f1000research.22660.1

**Published:** 2020-03-31

**Authors:** Xu Han, Niurka Molina Águila, Haiyang Yu, C. Nivaldo Linares Pérez, C. María Eugenia Toledo Romaní

**Affiliations:** 1 Pedro Kourí National Institute of Tropical Medicine, Havana, Cuba; 2 Finlay Institute of Vaccines, Havana, Cuba

**Keywords:** Streptococcus pneumoniae, serotype, antimicrobial resistance, 13-valent pneumococcal conjugate vaccine, China

## Abstract

The World Health Organization (WHO) recommends that pneumococcal conjugate vaccines (PCVs) be included in immunization programs worldwide. In China, the 7-valent pneumococcal conjugate vaccine (PREVNAR 7®) was authorized in 2008 but was not included in the national immunization programs. In 2016, PREVNAR 13®, a 13-valent pneumococcal conjugate vaccine (PCV13), was licensed for optional use in China. We will conduct a scoping review of the distribution of serotypes and antimicrobial resistance of *Streptococcus pneumoniae* in children aged under 5 years in China since the introduction of PCV13. We will obtain data from PubMed, the China National Knowledge Infrastructure (CNKI), and Wanfang Med Online. We will also review epidemiological data from WHO and the China Antimicrobial Surveillance Network (CHINET). Our analysis will include the condition of interest, the intervention, and the geographical region. All types of studies will be eligible for inclusion in the study database if they meet the inclusion criteria. This scoping review is intended to outline how *S. pneumoniae *serotypes are distributed, and it will map their antimicrobial resistance in children aged under 5 years in China. The results of this study will provide useful information on the impact of PCV13 in China.

## Introduction

Pneumonia accounts for 15% of all deaths of children under 5 years old, killing 808,694 children in 2017; *Streptococcus pneumoniae* is the most common cause of bacterial pneumonia in children ^1^. Although the widespread use of PCVs has decreased the burden of invasive pneumococcal disease (IPD) ^2^, *S. pneumoniae* is still a substantial issue because immunization causes serotype changes, the available PCVs are costly, and widespread antibiotic use during the last decades has resulted in drug-resistant strains of *S. pneumoniae* ^3^. The introduction of pneumococcal vaccines has decreased the incidence of drug-resistant strains in some areas ^4^. However, because of the widespread abuse of antibiotics and limited use of vaccines in many Asian countries, antibiotic resistance remains a very serious problem in Asia.

In China, pneumococcal disease is a major cause of morbidity and mortality in children ^5, 6^. Although PCV7 was licensed in China in 2008, it was not included in the national immunization program. The coverage rate of this vaccine is low; only 10% of Chinese children received PCV7 in 2016. ^7^ An analytical decision model indicated that the use of PCV7 could have prevented more than 16.2 million cases of pneumococcal disease and 709,411 deaths in China during the 10 years after the vaccine’s introduction ^8^. Similarly, although PCV13 was licensed for optional use in November 2016, it is not widely used because of low awareness and the high price of the vaccine.

China still lacks a systematic surveillance program for pneumococcal diseases. To date, studies analyzing serotypes and antimicrobial resistance of *S. pneumoniae* have not specified the date of introduction or the type of PCV. This study may provide useful information on the impact of PCV13 in China.

## Objectives

In this study, we aim to investigate how *S. pneumoniae* serotypes are distributed and to map antimicrobial resistance in children under age 5 years in China since the introduction of PCV13. We formulated our main review question using the SPICE framework (setting, perspective, intervention, comparison, evaluation). The framework and key elements are summarized in Table 1.

**Table 1.** Overview of the key elements of the review question.

Key Element	Elaboration
Setting	In mainland China, PCV13 has been licensed for optional use since November 2016.
Perspective	Children under the age of 5 years in China may benefit from vaccination.
Intervention	Individual or group interventions that include the PCV13 vaccine.
Comparison	Subgroups with and without the intervention and populations before and after the intervention.
Evaluation	Impact in terms of *S. pneumoniae* serotype distribution and antimicrobial resistance

## Methods

### Inclusion criteria

All types of studies are eligible for inclusion in our review if they meet all 5 of the following criteria: (1) Analyze serotype distribution or antimicrobial resistance to *S. pneumoniae* in China; (2) contain information on the impact of PCV13 in China; (3) include only subjects less than 5 years of age; (4) be written in English or Chinese; and (5) be published between November 2016 and February 2020.

Two investigators will independently evaluate the titles and abstracts of all articles according to the inclusion criteria. Any disagreement will be resolved through discussion among team members, and if there is difficulty related to the selection of studies, the criteria will be refined.

### Information sources

The study will begin in March 2020 and will include searches of PubMed, the China National Knowledge Infrastructure (CNKI), Wanfang Med Online, the WHO database, and the China Antimicrobial surveillance Network (CHINET).

PubMed is the largest database of medical information in the world. Created in 1946 by the National Library of Medicine, it includes the MEDLINE database, which contains information from more than 24 million articles published in 5,600 journals in 30 languages.

CNKI, as the largest, most-used online academic library in China, is a key national information construction project under the lead of Tsinghua University. It is the most comprehensive repository of knowledge in China, with more than 16 million website visits each day. In 2019, the website recorded 2.3 billion full-text downloads.

Wanfang Med Online aims to become a type of bridge that connects medical professionals and researchers in China with medical professionals and researchers around the world. At present, the English version includes about 200 selected Chinese medical journals including the top 115 medical journals published by the Chinese Medical Association, 1 Science Citation Index (SCI) journal, 7 Science Citation Index Expanded (SCIE) journals, and 60 Medline journals.

### Search

**Figure 1.**
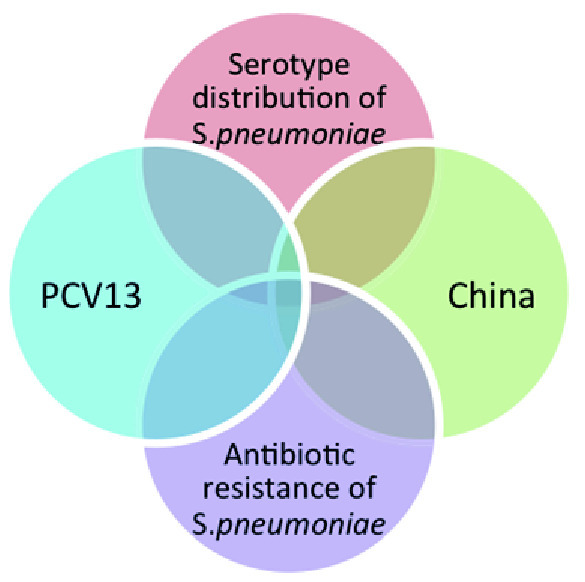
Summary of search strategy.

The search strategy will be based on a combination of three elements: the condition of interest, the specified intervention, and the geographical region ( Figure 1). The use of "OR" and "AND" to combine key words is shown in Table 2.

**Table 2.** Planned search syntax.

Keyword	Database
(((((“serotyping”[MeSH Terms]) OR “drug resistance”[MeSH Terms])) AND “streptococcus pneumoniae”[MeSH Terms]) AND 13-valent pneumococcal conjugate vaccine) AND China	PubMed
((serotype OR antibiotic resistance) AND streptococcus pneumoniae) AND (pneumococcal conjugate vaccine OR 13-valent pneumococcal conjugate vaccine)	CNKI
((serotype OR antibiotic resistance) AND streptococcus pneumoniae) AND (pneumococcal conjugate vaccine OR 13-valent pneumococcal conjugate vaccine)	Wanfang Med Online

### Study records

***Data management.*** Retrieved records will be automatically or manually exported into EndNote.

***Selection process.*** The search and selection process will be documented in a PRISMA flowchart ^9^. Screening and selection will be done in duplicate by two members of the review team. Any discrepancies discovered during screening of full-text papers will be discussed with the principal investigator.

***Data collection process.*** Two investigators will independently extract data from the studies that meet the inclusion criteria. Any disagreement will be resolved through discussion, and if they do not agree, the principal investigator will arbitrate. If data are missing or not sufficiently described in the studies, we will contact the corresponding author to obtain the missing information.

***Data items.*** The information technology department will develop two tables for organizing the data: a table that itemizes serotype, and a table that lists antimicrobial resistance to pneumococci. The tables will contain the elements listed in Table 3.

**Table 3.** Type of data to be collected.

Characteristics of extracted data	
Reference	Title, authors, publication type, journal, publication year
Study	Type of study, study period
Setting	Study objective, most frequent isolated serotypes and/or antimicrobial resistance pattern for *S. pneumoniae*
Population	Country where study took place; type and size of population undergoing intervention
Intervention	Description of intervention, and whether it was an individual or group intervention, or both
Study design	Elements needed to assess risk of bias as formulated by the authors
Evaluation	Evaluation of impact (the most predominant serotypes circulating in children, serotype coverage of the available PCVs, whether these serotypes contributed to the distribution of antibiotic-resistant isolates); denominator (children younger than 5 years of age)

### Outcomes and prioritization

Our review will focus on the scope of the available literature about the serotype distribution of and antibiotic resistance to *S. pneumoniae*, and the potential impact of PCV13 in China. We do not foresee outcome prioritization, but the approach will be descriptive.

### Risk of bias in individual studies

The risk of bias will be determined independently by two study team members. For nonrandomized studies, we will use the ROBINS-I tool ^10^, and for randomized trials, or interventions, we plan to use Cochrane Collaboration's risk-of-bias assessment tool ^11^.

### Data synthesis

A PRISMA flow chart and the methodological process will be described in detail for transparency. It will indicate the evidence identified and whether it was selected for inclusion in the review. If a study is excluded, the reasons will be provided.

The data will be summarized in the form of a figure, diagram, or table. A narrative summary of the findings will also be included. The strategy for synthesizing the data involves the use of analytical methods and will perform a descriptive synthesis of the data to map the distribution of serotypes and antimicrobial resistance of pneumococci in children under 5 years of age in China after the introduction of PCV13.

### Review team and roles

The review team is presented in Table 4.

**Table 4.** Review team, affiliations and roles.

Name	Affiliation	Role
Xu Han	Pedro Kourí Institute, Havana, Cuba	Write draft protocol, search & select studies, extract & synthesis data, write draft review
Haiyang Yu	Pedro Kourí Institute, Havana, Cuba	Write draft protocol, search & select studies, extract & synthesis data, write draft review
María Eugenia Toledo Romaní	Pedro Kourí Institute, Havana, Cuba	Write draft protocol, give methodological input, solve discordances in data extraction, write draft review, corresponding author
Niurka Molina Águila	Pedro Kourí Institute, Havana, Cuba	Provide context knowledge, give methodological input
Nivaldo Linares Pérez	Finlay Institute of Vaccine, Havana, Cuba	Provide topic expertise, interpret findings, give feedback on draft texts

### Study status

This review is a part of ongoing research in China on the interchangeability of PCV13 with the Cuban vaccine PCV7-TT. When we complete this scoping review, we will consider conducting a systematic review, and we intend to publish the results in an international journal. While preparing the present protocol, we performed preliminary searches to get an idea of the size of the available literature.

### Ethics

Because the scoping review methodology consists of reviewing and synthesizing the data already published, this part of the study is not subject to ethical approval.

### Study strengths

- This systematic scoping review will be the first to map antimicrobial resistance to and distribution of pneumococcal serotypes in children less than 5 years of age in China after the introduction of PCV13.
- It will present an overview of all studies on serotype distribution and antimicrobial resistance, and the analysis will clarify the characteristics and possible differences between the provinces of China.
- A search of three large databases will enable us to create a comprehensive map of antimicrobial resistance to and distribution of pneumococcal serotypes in China.

## Data availability

No data are associated with this article.
