# Supplementary material for: Serotypic distribution and antimicrobial resistance of Streptococcus pneumoniae in Chinese children under 5 years after the introduction of the 13-valent conjugate pneumococcal vaccine: protocol for a scoping review
Source: F1000Res. 2020 Mar 31;9:221. [Version 1] doi: 10.12688/f1000research.22660.1 (PMC7400691; doi:10.12688/f1000research.22660.1)
Supplement: Peer review history

# Peer review: Reviewer response for version 1

**DOI:** 10.5256/f1000research.25017.r66175

**Published:** 2020-07-30

**Authors:** Eric Bonnet

**Affiliations:** 1 Institute of Genomics, CEA (French Alternative Energies and Atomic Energy Commission), Evry, France

In this article, the authors describe a protocol for a scoping review of the distribution of serotypes and antimicrobial resistance of *Streptococcus pneumoniae* in children aged under 5 years in China since the introduction of the 13-valent conjugate pneumococcal vaccine (PCV) in China in 2016.

 Pneumonia accounts for quite a large number of deaths for children under 5 years old worldwide and *Streptococcus pneumoniae* is the most common cause of the disease. In China, approximately 30,000 children die of pneumonia each year. The introduction of PCV has quite dramatically reduced the impact of the disease in many areas of the world. However, infections by *S. pneumoniae* are still a problem because of the many serotypes for this bacteria and also because immunization triggers serotype changes. In China, PCVs were introduced quite recently (2008), with only 10% of the children that received the vaccine. Furthermore, there is no systematic surveillance program for pneumococcal diseases. Therefore, the main goal of the protocol described in this study is very timely and will be very useful for researchers and epidemiologists.

 The article is well written, concise, and with enough precision. The authors focus on the PCV 13-valent which was introduced in China in 2016, and they intend to analyze all the relevant literature published on the subject till February 2020. The criteria defined for the selection of the articles are well described and relevant. The authors intend to use three main databases to conduct their survey, namely PubMed, the China National Knowledge Infrastructure (CNKI) and Wanfang Med Online. Review epidemiological data will also be included from the World Health Organization and the China Antimicrobial Surveillance Network (CHINET).

 I only have a few remarks that could help to improve further the quality of the manuscript:

 In the methodology section, the authors mention a potential “risk of bias”, but do not elaborate much on that topic. I would suggest explaining what this risk is about and if possible give one or two concrete examples.

 It seems that a couple of studies have investigated with simulations of the cost-effectiveness of PCV-13 usage in China. See for instance Mo *et al. *Pediatr. Infect. Dis. J. 2016 PMID 27753771, Tyo *et al. *Vaccine 2011 PMID 21745516. ^1^^,^^2^ I would suggest to lookup for more publications of this type and to include a small paragraph summarizing the results in the introduction.

 Maybe it would also be useful to include a small paragraph on the serotype distribution in China before the introduction of PCV-13, see Lyu *et al.* Expert Rev. Vaccines PMID 28745918. ^3^

Is the study design appropriate for the research question?

Yes

Is the rationale for, and objectives of, the study clearly described?

Yes

Are sufficient details of the methods provided to allow replication by others?

Yes

Are the datasets clearly presented in a useable and accessible format?

Not applicable

Reviewer Expertise:

bioinformatics, biology, immunology

I confirm that I have read this submission and believe that I have an appropriate level of expertise to confirm that it is of an acceptable scientific standard.

## References

[1] : Cost-effectiveness and Health Benefits of Pediatric 23-valent Pneumococcal Polysaccharide Vaccine, 7-valent Pneumococcal Conjugate Vaccine and Forecasting 13-valent Pneumococcal Conjugate Vaccine in China. *Pediatr Infect Dis J*.35(11) : 10.1097/INF.0000000000001288 e353-e361 10.1097/INF.0000000000001288 27753771

[2] : Cost-effectiveness of conjugate pneumococcal vaccination in Singapore: comparing estimates for 7-valent, 10-valent, and 13-valent vaccines. *Vaccine*.2011;29(38) : 10.1016/j.vaccine.2011.06.091 6686-94 10.1016/j.vaccine.2011.06.091 21745516

[3] : A systematic review about Streptococcus Pneumoniae serotype distribution in children in mainland of China before the PCV13 was licensed. *Expert Rev Vaccines*.16(10) : 10.1080/14760584.2017.1360771 997-1006 10.1080/14760584.2017.1360771 28745918

# Peer review: Reviewer response for version 1

**DOI:** 10.5256/f1000research.25017.r62038

**Published:** 2020-04-09

**Authors:** Guanlan Zhao

**Affiliations:** 1 Department of Community Nursing, Preventive Medicine and Public Health and History of Science, University of Alicante, Alicante, Spain

The authors describe in details the highlights of this study protocol. Pneumococcal disease is one of the main causes of morbidity and mortality in children under 5 years old. In 2018, the number of newborns in China exceeded 15 million, and there is a huge clinical demand for the vaccine. In China, there are two types of vaccines. The first type of vaccine refers to the vaccine provided by the government to public free of charge, and the public shall be vaccinated in accordance with the government's regulations, mainly with the routine immunization vaccine for children and the second type of vaccine refers to the vaccine that is provided by the public at their own expense and voluntarily, such as Streptococcus pneumoniae vaccine. Due to the huge burden of disease and the acknowledgement of protecting the children’s’ health about pneumococcal disease, it also shows the importance and feasibility of this study. I think it be useful to create a comprehensive map of this topic in China.

 About information sources, the author chooses the China National Knowledge Infrastructure (CNKI), Wanfang Med Online, there are two important Chinese databases. I believe that the integration of these database together with Pubmed will make the future scoping review more complete and perfect.

 Possible recommendation of future scoping review: In recent years, China's economy has developed rapidly, but the gap of economic level still exists in different regions of China, and the distribution of medical resources will also be affected. Is there known to be differences in education levels or income levels in selected articles? It might be good to discuss what possible other socioeconomic differences might exist in geographical regions that would account for differences in the vaccination rate in the scoping review.

 The authors describe the objective and material and methods of study clearly.

Is the study design appropriate for the research question?

Yes

Is the rationale for, and objectives of, the study clearly described?

Yes

Are sufficient details of the methods provided to allow replication by others?

Yes

Are the datasets clearly presented in a useable and accessible format?

Yes

Reviewer Expertise:

Epidemiology, Occupational Health, Cohort Studies

I confirm that I have read this submission and believe that I have an appropriate level of expertise to confirm that it is of an acceptable scientific standard.

# Response

**Published:** 2020-04-12

**Authors:** Xu Han

**Affiliations:** Pedro Kouri Tropical Medicine Institute, China

Thank you for your comments and suggestions!

We agree with your comments, it is very interesting that you mentioned about these factors affecting immunization coverage level in China, especially the impact caused by the socio-economic differences between the geographic regions.
